# Dear Readers, Authors, Reviewers and Editorial Board Members of *Advances in Cognitive Psychology*

**DOI:** 10.5709/acp-0181-4

**Published:** 2016-03-31

## Abstract

In this first newsletter of 2016 we first wanted to wish all our readers, authors, reviewers, and editorial board members a happy, healthy, inspiring, but also peaceful new year. Recently, the fourth issue of Advances in Cognitive Psychology of 2015 was completed, which is of course freely available on our website. The current newsletter will also be included in the first issue of 2016.

At the end of the last year, we decided to update our editorial board as some of our members indicated that due to other duties they no longer could find sufficient time to properly carry out their editorial duties. Our editorial board now consists of Elger Abrahamse, Arndt Broeder, Claus-Christian Carbon, Simone Dalla Bella, Małgosia Fajkowska, Markus Kiefer, Iring Koch, Grzegorz Króliczak, Michał Kuniecki, Markus Lappe, Bernd Leplow, Severine Samson, Ingrid Scharlau, Elisabet Service and Rolf Verleger. We want to thank the resigning editorial members for the work that they carried out in support of the journal: Gisa Aschersleben, Christian Büchel, Anna Grabowska, Peter Keller, Bruno Repp, Michael Spivey, Kate Stevens, Martin Voracek, Dirk Vorberg, and Vincent Walsh. 

What’s new in ACP? Given our inclusion in the ISI-list of journals and the publication of ACP’s first Impact Factor (1.646), the Polish Ministry of Science and Education recently decided to move our journal from the B-list to the A-list of scientific journals, and they also indicated that publication in our journal will now be evaluated with a score of 20 points instead of 10 points. This implies that it becomes very attractive for Polish scientists to publish in our journal. We of course look forward to receiving their submissions.

In 2015, the Neuronus conference took place in Kraków, and in this year a special issue will be published with several contributions from this conference. We would again like to draw your attention to the Neuronus conference (http://neuronusforum.pl/). This year, the conference will be held in Kraków from the 22nd to the 24th of April 2016. 

On our website, we also listed all of the reviewers that were consulted in 2015: You played a key role in keeping our high standards, and special thanks go to you! Out of all of ACP’s submissions in 2015, 19% were rejected without external review, 44% were rejected after external review, 20% were accepted, and the remaining manuscripts are currently being revised or are under review. 

In the last year, there were no major changes in the journal’s budget. We are very grateful to the University of Finance and Management in Warsaw that continued to financially support ACP in the last year. We also submitted a proposal to get further support by the Polish Ministry of Science and Education. Nevertheless, there remains the possibility that we will have to ask authors for (voluntary) contributions to cover ACP’s publication costs in the future. 

In conclusion of this letter, we kindly wanted to remind you that we continue to welcome your submissions to the journal. Also, should you have any questions or comments regarding this letter, or ACP please feel always free to contact us. 

Sincerely yours,

Rob van der Lubbe

Ulrich Ansorge

